# Supplementary figures and images for: Mutational analysis of human norovirus VP2 elucidates critical molecular interactions for virus assembly
Source: J Virol. 2025 Dec 30;100(2):e01420-25. doi: 10.1128/jvi.01420-25 (PMC12911861; doi:10.1128/jvi.01420-25)

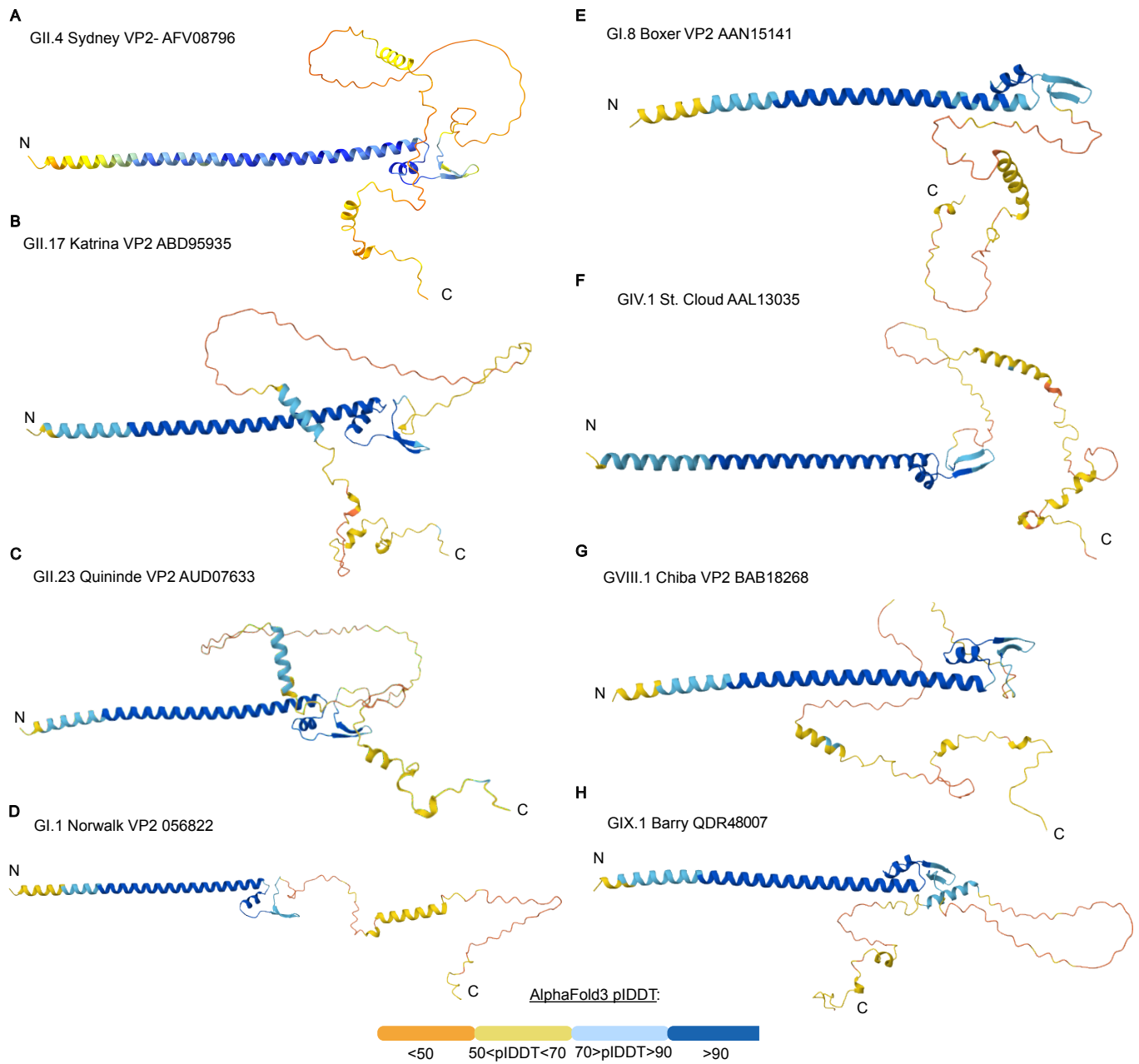

**Fig. S1**

A

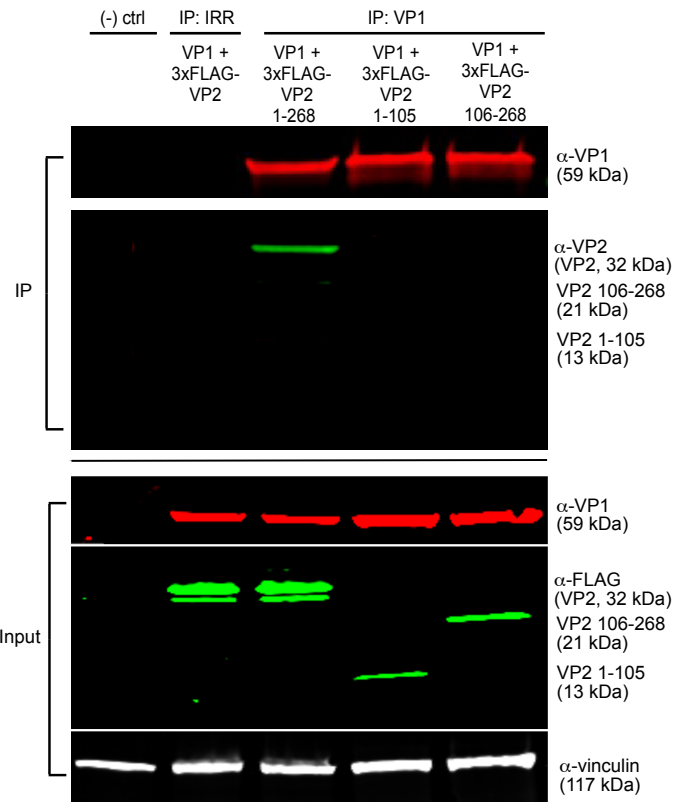

B

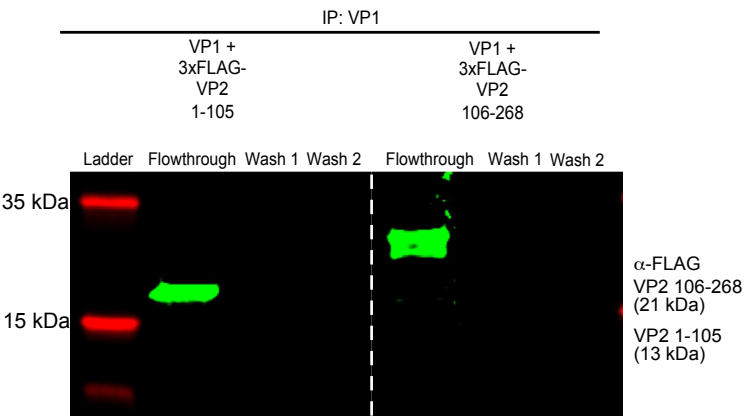

Fig. S2

**A** GII.4 Sydney VP2 AFV08796

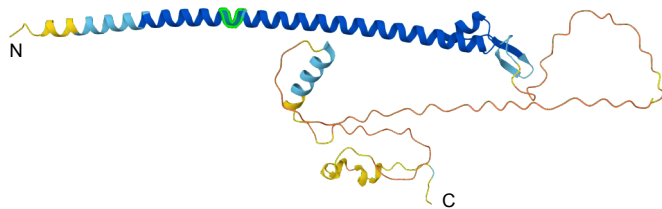

**B** GII.4 Sydney VP2 40-EEEE-43

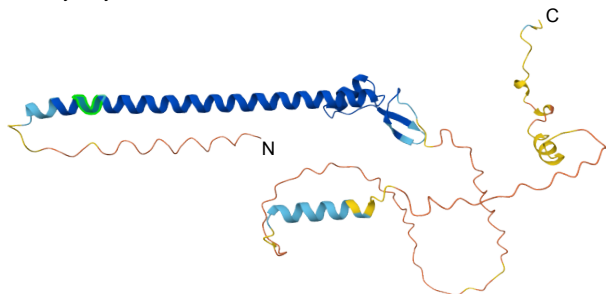

**C** GII.4 Sydney VP2 40-AAAA-43

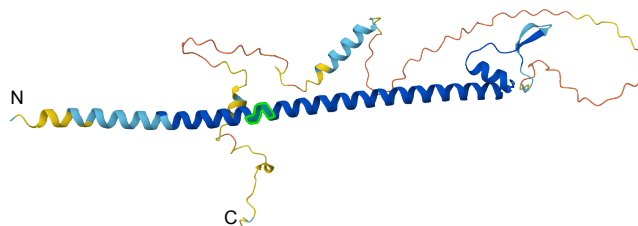

AlphaFold3 pLDDT:

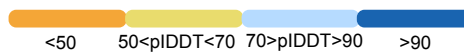

**Fig. S3**

Supplement: Supplemental figures — Fig. S1 to S3. [file jvi.01420-25-s0001.pdf]
