# Supplementary material for: Mutational analysis of human norovirus VP2 elucidates critical molecular interactions for virus assembly
Source: J Virol. 2025 Dec 30;100(2):e01420-25. doi: 10.1128/jvi.01420-25 (PMC12911861; doi:10.1128/jvi.01420-25)
Supplement: Supplemental legends — Legends for Fig. S1 to S3. [file jvi.01420-25-s0002.docx]

**Supplementary Figure Legends**

**Fig. S1. Alphafold3 predicted structures of HuNoV VP2 from representative genogroups.** Sequences were selected from at least one representative genogroup, including (**A**) GII.4 Sydney (NCBI accession #AFV08756), (**B**) GII.17 Katrina (#ABD95935), (**C**) GII.23 Quininde (#AUD07633), (**D**) GI.1 Norwalk (#056822), (**E**) GI.8 Boxer (#AAN15141), (**F**) GIV.1 St. Cloud (#AAL13035), (**G**) GVIII.1 Chiba (#BAB18268), and (**H**) GIX.1 Barry (#QDR45007). For each VP2 model, the N- and C-termini are labeled. Structures are colored by plDDT confidence scores: blue (plDDT > 90, high confidence), cyan (70 < plDDT ≤ 90), yellow (50 < pLDDT ≤ 70), and orange (plDDT ≤50, low confidence). The predicted α-helical N-terminal domains are consistently modeled with high confidence, while the C-terminal regions are more variable in length and secondary structure and often predicted to be disordered. Representative genogroups based on designation by *Chhabra* *et al*. (6).

**Fig. S2. Analysis of truncated VP2 interaction with VP1.** Western blot analysis of wild-type VP1, 3xFLAG-VP2 and truncated VP2 AA 1-105 or 106-268 interaction via co-expression and co-immunoprecipitation. The proteins indicated above each lane were co-expressed and co-immunoprecipitated using α-VP1 mAb. Each protein was detected using the antibody indicated to the right of each immunoblot as described in **Fig. 3B-3C** (**B**) Immunoprecipitated fractions including flowthrough, wash 1 and wash 2 were analyzed for VP2 as VP2 truncated variants did not pull down with VP1 and VP2 is seen in the flowthrough but not in the wash fractions.

**Fig. S3. Alphafold3 predicted structures of wild-type HuNoV VP2 or recombinant VP2 mutated at AA 40-43.** (**A-C**) AlphaFold3 predicted structure of VP2 with N- and C-termini labeled. AA 40-43 are highlighted (green) in each predicted structure. (A) Predicted structure of wild-type VP2. (**B**) Predicted VP2 structure with AA 40-43 mutated to 4x glutamate and (**C**) AA 40-43 mutated to 4x alanine. The α-helical N-terminal domains at mutation site are consistently modeled with high confidence, while the C-terminal regions are more variable in length and secondary structure.
